# Towards unbiased skin cancer classification using deep feature fusion

**DOI:** 10.1186/s12911-025-02889-w

**Published:** 2025-01-31

**Authors:** Ali Atshan Abdulredah, Mohammed A. Fadhel, Laith Alzubaidi, Ye Duan, Monji Kherallah, Faiza Charfi

**Affiliations:** 1 National School of Electronics and Telecoms of Sfax, University of Sfax, Sfax, Tunisia; 2 College of Computer Science and Information Technology, University of Sumer, Thi-Qar, Iraq; 3 School of Mechanical, Medical, and Process Engineering, Queensland University of Technology, Brisbane, Australia; 4 School of Computing, Clemson University, Clemson, SC USA; 5 Faculty of Science of Sfax, University of Sfax, Sfax, Tunisia

**Keywords:** Deep learning, Skin cancer classification, Transfer learning, Explainable AI, Grad-CAM, Feature fusion

## Abstract

This paper introduces SkinWiseNet (SWNet), a deep convolutional neural network designed for the detection and automatic classification of potentially malignant skin cancer conditions. SWNet optimizes feature extraction through multiple pathways, emphasizing network width augmentation to enhance efficiency. The proposed model addresses potential biases associated with skin conditions, particularly in individuals with darker skin tones or excessive hair, by incorporating feature fusion to assimilate insights from diverse datasets. Extensive experiments were conducted using publicly accessible datasets to evaluate SWNet’s effectiveness.This study utilized four datasets-Mnist-HAM10000, ISIC2019, ISIC2020, and Melanoma Skin Cancer-comprising skin cancer images categorized into benign and malignant classes. Explainable Artificial Intelligence (XAI) techniques, specifically Grad-CAM, were employed to enhance the interpretability of the model’s decisions. Comparative analysis was performed with three pre-existing deep learning networks-EfficientNet, MobileNet, and Darknet. The results demonstrate SWNet’s superiority, achieving an accuracy of 99.86% and an F1 score of 99.95%, underscoring its efficacy in gradient propagation and feature capture across various levels. This research highlights the significant potential of SWNet in advancing skin cancer detection and classification, providing a robust tool for accurate and early diagnosis. The integration of feature fusion enhances accuracy and mitigates biases associated with hair and skin tones. The outcomes of this study contribute to improved patient outcomes and healthcare practices, showcasing SWNet’s exceptional capabilities in skin cancer detection and classification.

## Introduction

The most important organ of the human body is the skin. It protects the body from extreme temperatures, UV rays, and chemicals and acts as a waterproof shield. The abnormal growth of skin cells may cause skin cancer [1]. It occurs mainly outside the body, but can extend to other areas, including the eyes, nose, and neck [2]. The epidermis, which consists of layers, is usually the target of such malignant disease [3]. Skin cancers begin as precancerous lesions that are not malignant but become malignant over time. This disease has many causes that may sometimes lead to death. Therefore, early detection, regular skin examination procedures, correct diagnosis, and treatment are the keys to preventing skin cancer [4–6]. Skin cancer is typically detected by doctors who identify suspicious areas on the skin. However, recent studies [7–9] have shown that the use of advanced artificial intelligence techniques, such as Convolutional Neural Networks (CNN), can aid doctors diagnose diseases earlier stage [10]. This has inspired researchers to develop advanced technological tools for diagnosing skin cancer diseases.

The diagnosis of skin cancer involves various methods such as visual examination, dermoscopy, and biopsy. Dermoscopy and the expertise of a physician have significantly improved the accuracy of the identification. However, manual diagnosis poses challenges, prompting the adoption of computer-assisted diagnosis (CAD) when expert consultation is limited [11, 12]. Machine learning (ML), particularly deep learning utilizing convolutional neural networks (CNNs), has transformed the classification of skin cancer. Traditional ML methods are less prevalent, with deep learning models as effective as dermatologists in image recognition. Despite challenges like insufficient training data, deep learning improves accuracy, with a 15–20% enhancement in cancer prediction over the past two decades [13]. While some deep CNN models increase processing costs, integrating ML into computer-aided design (CAD) systems improves tumour disease identification and treatment, making the process more cost-effective. The fusion of machine learning and computer vision in CAD systems significantly enhances skin cancer detection, particularly in its early stages [14]. AI in medicine is gaining prominence for skin cancer diagnosis, providing swift, precise, and consistent disease recognition. Computer-aided design streamlines the identification and treatment of tumour diseases, complementing traditional imaging methods like MRI, PET, and X-rays. AI-driven advancements seek to enhance outcomes and mortality rates by improving the early detection of skin cancer, addressing the constraints of subjective and time-consuming diagnostic procedures [15].

The following is a summary of the numerous intriguing observations revealed by our contributions.

1. Developed a robust Deep Convolutional Neural Network, SWNet, from scratch, emphasizing network width expansion and global average pooling. Established the efficacy of the architecture by incorporating discrete layers of features at each stage, enhancing feature fusion.
2. Implementation of SWNet algorithm for skin cancer classification achieved a 99.95% F1-score distinguishing between benign and malignant cases, surpassing the CNN model.
3. Explainable Artificial Intelligence (XAI): We applied XAI techniques to interpret model outcomes. Enhanced understanding and confidence in results through XAI, contributing to the transparency of the model’s decision-making process.
4. We conducted benchmarking tests, evaluated model robustness, and applied bias mitigation techniques for skin cancer classification. We used fine-tuned and pre-trained EfficientNet, MobileNet, and Darknet models as benchmarks for comparison. Our results showed that SWNet outperformed the other models, highlighting its effectiveness in the field. We also demonstrated the significance of feature fusion in mitigating biases. We assessed the robustness of our models across diverse datasets, including HAM10000 and ISIC2019_2020. These tests established the reliability of SWNet and its contribution to bias reduction through feature fusion.

The article is systematically structured, starting with a discussion related to the work in the field in “Related works” section. This sets the contextual backdrop for the proposed research. “Proposed methodology” section comprehensively explains the method employed in this study, elucidating the novel approach and methodology applied. “Experimental result” section presents the results, revealing the results and insights gleaned from implementing the proposed method. Finally, “Limitations and future directions” section concludes the article by offering conclusions drawn from the study and providing recommendations for further avenues of research in the domain. This organization ensures a logical flow of information.

## Related works

Although non-automated medical communication systems have shown impressive results, assessing a patient’s condition still requires the presence of professional medical experts. There is a clear and pressing need for automatic skin cancer detection. In this section, we will discuss the literature related to skin cancer detection and classification using machine learning and deep learning.

In [16], CNN techniques and SVM and KNN machine learning classifiers were applied for image feature extraction to show the skin lesion image’s borders, texture, and color. The accuracy rates for the SVM and KNN classifiers were 77.8% and 57.3%, respectively. When using deep learning, the accuracy grows up to 85.5%. Despite the excellent result, splitting the image into parts can miss some relevant information to predict the class correctly. In another study, A. Demir et al. [17] employed the ResNet-101 and Inception-v3 transfer learning models. Additionally, they implemented a data augmentation approach to address the issue of overfitting that may arise from training the model on a limited dataset. The accuracy achieved by the models was 84.09% and 87.42%, respectively. T. Emara et al. [18] used the Inception V4 model pre-trained on ImageNet on the HAM10000 dataset to diagnose skin cancer disorders. The data set displays an imbalance, which led to the use of a data sampling strategy to address this problem. Their model has a rating accuracy of 94.7%.

In 2020, C.N. Vasconcelos et al. [19] trained the ISBI 2016 dataset with GoogLeNet. The researchers used standard data augmentation during sample processing to address the issue of an imbalanced training dataset influencing CNN performance. Maximum accuracy was 83.6%. Qasim et al. [20] used the same model to leverage knowledge transfer effectively to classify eight distinct categories of skin lesions in the ISIC 2019 dataset. The model achieved a level of accuracy of 94%. Hosny et al. [21] their method based on deep convolutional neural network (DCNN) for skin image classification. The methodology includes pre-processing to segment regions of interest (ROIs), augmenting ROI images with rotations and translations, and using different DCNN architectures. Deep convolutional neural networks (DCNNs) replace the last three layers to improve lesion classification. Three datasets were used to evaluate and fine-tune this technique. GoogleNet performed well on the MEDNODE, DermIS & DermQuest, and ISIC 2017 datasets, achieving classification accuracies of 99.29%, 99.15%, and 98.14%, respectively. The model may miss important information when medical images are segmented. Diame et al. [22] Classified seborrheic keratosis and melanoma using three deep-learning models: DenseNet161, Inception-v4, and ResNet-152. These models’ accuracy was 86.3%, 82.0%, and 88.7%.

In 2022, Ahmadi et al. [23] pre-trained the Inception-ResNet-v2 CNN on 57,536 lesions. Pre-training helped the model identify melanoma in lesions. For classification, the model included patient-specific parameters, particularly lesion location, age, and sex, in addition to the lesion image. The model accuracy was 94.5%. Also, Li, Z. [24] Used the pre-trained technique to transfer learning Inception-ResNetV1 with the SVM classifier; the model was tested on ISIC 2019. A Classification accuracy rate of 98% helps diagnose clinical melanoma.

In 2023, K. Mridha, J. Shin, et al. [25] developed a deep learning (DL) prediction model for melanoma classification on the HAM10000 dataset, and using a data augmentation technique, they also used the resulting model interpretation technique (Grad-CAM, Grad-CAM++) with 82% classification accuracy. Thanka, M. Roshni, and colleagues [26] used the VGG16 model as a transfer learning technique for feature extraction. These features are subsequently fed into the XGBoost classifier and optical gradient boosting machine for severity assessment and classification of benign and malignant conditions. The integration shows an accuracy level of 99.1%. The XGBoost and LightGBM models achieve a classification accuracy of 99.1% and 97.2%, respectively.

In 2023, B. Tasar presented a modified CNN framework that employed transfer learning to categorize skin lesions in dermoscopy images. The model utilized imageNet-pre-trained CNN architectures and underwent training on the HAM10000 skin lesions dataset. The classification accuracy for the modified DenseNet121, VGGNet16, ResNet50, MobileNet, and Xception models was 94.29%, 93.28%, 87.10%, 83.10%, and 80.05%, respectively. These findings suggest that the proposed transfer learning framework surpassed the performance of traditional deep learning architectures in classifying skin lesion types [27].

M. Tahir et al. [28] suggested DSCC_Net, a CNN-based deep-learning network for classifying melanoma. Three data sets were used to evaluate it. They struggled with the problem of an unequal distribution of categories across the data set. DSCC_Net performs better than six core models (ResNet-152, Vgg-16, Vgg-19, Inception-V3, EfficientNet-B0, and MobileNet), with accuracy scores of 94.17%, 93.76% retrieval, 94.28%, and 93.93%. F1 among the four categories of skin cancer.

G. Qasim et al. [29] used Deep spiking neural networks with surrogate gradient descent to classify 3670 ISIC 2019 melanoma pictures and 3323 non-melanoma photos. Spiking VGG-13 outperformed both VGG-13 and AlexNet models with an accuracy of 89.57% and an F1 score of 90.07%. This improvement occurred with less trainable parameters. The study by S. Waheed et al. [30] utilized dermoscopy images to demonstrate the application of a deep learning system for melanoma identification. The researchers achieved an F1 score of 90.87% and sensitivity, specificity, and precision scores of 92.46%, 92.23%, and 92.46%, respectively. In another study, Y. Dahdouh et al. [31] used a watershed algorithm in their proposal that combines deep learning and reinforcement learning techniques to detect and classify skin cancer. The proposed system achieved up to 80% accuracy on the HAM10000 data set. Using a watershed algorithm for image segmentation may only capture relevant image information if image gradation is calculated to identify potential regions of interest. It can also lead to excessive fragmentation if not used carefully.

R. Maalej et al. [32] used Mobilenet as a transfer learning model to extract features for classifying breast cancer histopathological images. This model was trained on the Breakhis dataset, and they treated the data imbalance using a data augmentation technique; the accuracy of the proposed model reached 90.0%.

### Deep learning

**Fig. 1:**
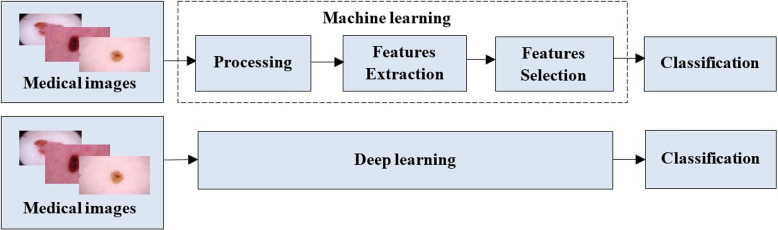
Illustrating the difference between ML and DL

Recently, the domain of deep learning has demonstrated significant efficacy in solving a range of issues related to pattern identification and the field of computer vision, including image classification [33, 34]. This technique has been effectively showcased in numerous biomedical image analysis challenges. Classical machine learning involves a series of methodologies that require pre-processing and identifying relevant features from input data, such as extracting features like texture and intensity and forming descriptors from images meticulously. Subsequently, the models are trained to generate predictions using features. However, this methodology is limited by the fact that it requires domain expertise for the selection of features. A process that can be laborious and may not exhaustively extract intricate patterns within complex medical images [35]. Figure 1 illustrates the distinction between deep learning and machine learning.

On the other hand, the significant advancement of deep learning networks, a branch of machine learning that utilizes neural networks, particularly those with intricate structures comprising numerous layers, has facilitated the automated acquisition of characteristics from unprocessed data. Convolutional neural networks, a prevalent kind of deep learning, demonstrate exceptional proficiency in analyzing medical pictures through their ability to acquire significant information directly from the images hierarchically. Deep learning models can have the capability to identify intricate spatial and contextual details through an end-to-end learning process. This leads to enhanced precision and reliability in making predictions and managing complex and vital tasks such as image assimilation at different scales [35]. Implementing this approach has brought about a significant transformation in medical image analysis. It has effectively minimized the reliance on subjective and manually crafted features, leading to a substantial enhancement in diagnostic precision and the ability to detect diseases. Nevertheless, this challenge still exists due to various factors, including low image resolution, overlapping elements, intricate shapes, etc.

**Fig. 2:**
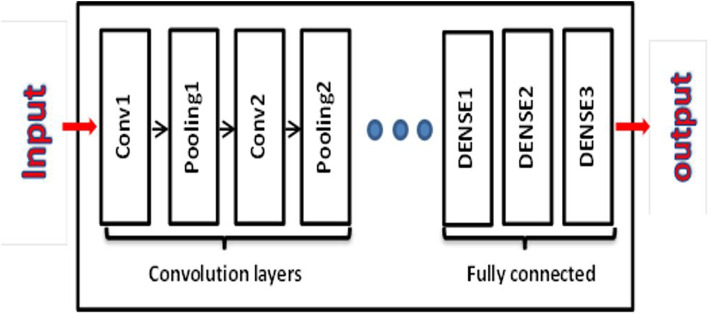
The basic structure of CNN

In summary, traditional machine learning methods in medical image analysis are heavily dependent on manual features and traditional algorithms. In contrast, deep learning techniques, particularly Convolutional Neural Networks (CNNs), autonomously acquire hierarchical features directly from raw images. This characteristic of deep learning contributes to enhanced accuracy and performance in medical image analysis. The primary distinction resides in the automation of feature extraction and representation, rendering deep learning highly advantageous for collecting nuanced patterns seen in intricate medical images [36]. A CNN is a feed-forward neural network that, as depicted in Fig. 2, consists of one or more convolutional layers, followed by pooling layers.

AlexNet is a prominent CNN because it was first developed in 2012 [37]. This development includes many improvements. First, I used rectified linear unit (ReLU) activation to increase the non-linearity of the network, which helped solve gradient descent problems. To avoid overfitting, it also applied a dropout technique, similar to regularization, which involves stochastic activation and deactivation of neurons across all layers. By activating neurons in various ways, we can force data down new paths, improving the network’s generalization ability. Finally, data augmentation strengthens the network by emphasizing image properties rather than the images themselves. It is implemented by providing images that have been arbitrarily cropped, rotated, and translated before being fed into the network. Finally, additional convolutional layers are placed before pooling layers to improve classification accuracy.

Afterwards, the VGG16 network was introduced, with some improvements included [38]. It has improved the depth of the network, allowing it to learn more complex features. It also used 3 x 3 convolutional filters and a maximum of 2 x 2 pooling layers throughout the network. This simplicity made it easy to understand and iterate. Small convolutional filters enabled it to capture more detailed features in early layers. It also used dropout regulation, which helped reduce overfitting by randomly turning off a portion of a neuron during training. A deep convolutional neural network architecture is GoogleNet. It was introduced in 2014 and used numerous parallel convolutional layers using different filter sizes to capture features in photos at various scales. This framework architecture makes image recognition tasks more accurate and efficient. ResNet (Residual Network) is a deep neural network architecture that uses skip connections to solve the problem of vanishing gradients and make very deep network training possible [39]. The DenseNet network has been designed to incorporate a highly intricate structure wherein the blocks of layers are interconnected [40]. However, MobileNet is a lightweight convolutional neural network model for efficient inference on mobile and embedded devices [37]. The networks mentioned above have been developed to categorize various medical image classification tasks efficiently [41]. We aim to design an extended convolutional neural network to take different features from multiple levels and integrate them at each network stage, facilitating efficient gradient propagation and bringing significant benefits.

## Proposed methodology

Early skin cancer detection can save patients’ lives and increase their chances of survival. This section contains several steps, which are described below.

###  Data gathering

**Fig. 3:**
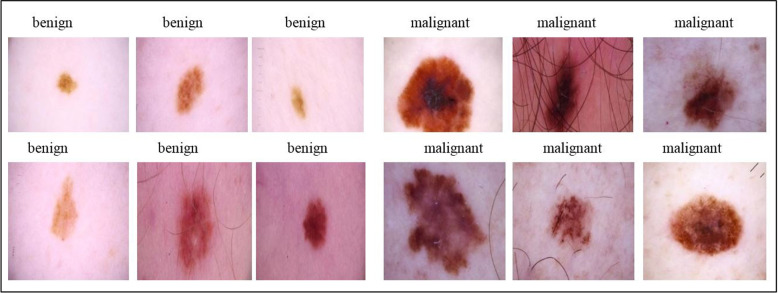
Samples of patches with labels from the dataset

Data acquisition includes the data collection process obtained via the electronic platform. We used four data sets containing images of skin cancer, divided into benign and malignant categories. The first dataset is taken from (https://www.kaggle.com/datasets/hasnainjaved/melanoma-skin-cancer). The skin cancer melanoma dataset contains 10,605 images. Skin cancer of melanoma is a deadly cancer. This data set will be useful for developing deep learning models for accurate classification of melanoma. Images were 300 pixels on the longest side and saved in JPEG file format. Figure 3 shows a sample of the data set. The second dataset is from the International Skin Imaging Collaboration (ISIC) (https://www.kaggle.com/datasets/ nodoubttome/skin-cancer9-classesisic). This collection consists of 2357 images of malignant and benign tumours. The third dataset is from Skin Cancer MNIST: HAM10000 (https://www.kaggle.com/datasets/kmader/skin-cancer-mnist-ham10000), which is a folder named HAM10000_images_part_1 containing a set of images with names. An Excel file containing data related to those images is in the folder. We extracted them from the folder so that the df, bkl, and nv images are in the DX column in Excel and put them in a folder on the desktop called Benign. Other images within the same dx column, namely mel, bcc, ak, and akiec, are also placed in a folder on the desktop that we call malicious. This collection consists of 10,015 images of benign and malignant tumors. The fourth dataset is from (https://www.kaggle.com/datasets/qikangdeng/isic-2019-and-2020-melanoma-dataset); this dataset is a set of melanoma images from the ISIC2019 and ISIC2020 challenge datasets; consisting of 11449 images for malignant and benign tumours. In this study, the same data are used. The dataset consists of images divided into two parts: 80% were used for training and 20% for testing. This study uses publicly available datasets. Per the dataset guidelines, no additional ethics approval is needed for secondary use.

### Data pre-processing

After collecting the preprocessing is done for the images received from the dataset. Thus, all images were resized to a uniform size of 224 pixels in width and 224 pixels in height before being input into the deep learning model,order to be compatible with the inputs of our proposed model and with the pre-trained model.

### Pre-trained CNN architectures

**Fig. 4:**
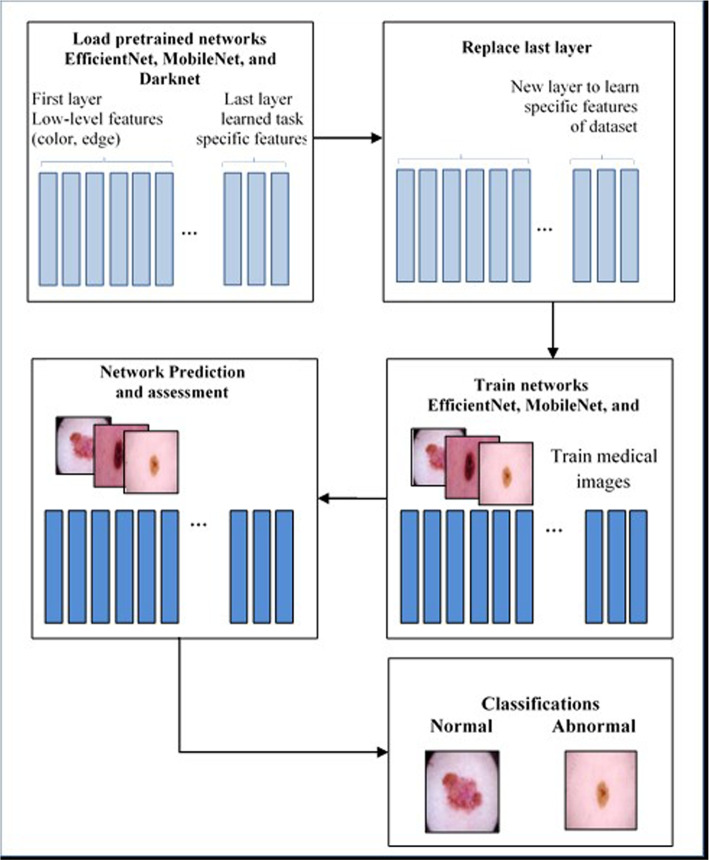
Procedures were performed using our method of knowledge transfer

Leveraging pre-trained convolutional neural networks (CNNs) offers the potential for refinement through fine-tuning with ImageNet, which is particularly advantageous in medical image datasets where expansive networks can adeptly learn task-specific features [42]. Extensive research [43] underscores the efficacy of transfer learning in augmenting the performance of medical image classification. In our exploration, we selected EfficientNet, MobileNet, and Darknet CNN models with careful consideration of the number of layers. The procedural stages of our transfer learning approach are outlined in Fig. 4.

EfficientNet, distinguished by a compound scaling method, dynamically adjusts its layer count based on model variants (e.g., B0, B1, B2). Introducing a balancing factor for efficient scaling of depth, width, and resolution, this architecture is celebrated for its outstanding performance with diminished parameters and computational demands, aligning seamlessly with the demands of medical image classification tasks.

DarkNet, initially crafted for YOLO (You Only Look Once) object detection, showcases an impressive array of 53 convolutional layers. Operating with an input size of 224x224x3, Darknet employs deep layers to capture intricate features, establishing itself as a compelling choice for the classification of skin pathology.

MobileNet, now equipped with 28 convolutional layers and maintaining a standardized input size of 224x224x3, optimizes computational efficiency through depthwise separable convolutions. This technique partitions regular convolutions into depth and pointwise layers, diminishing parameters and computational intricacies. MobileNet is particularly well-suited for the classification of skin pathology, especially in resource-constrained environments. Retraining these pre-existing networks on our dataset shifted our focus to discerning between normal and pathological skin conditions. The prominent instances of transfer learning for classification now encompass EfficientNet, MobileNet, and Darknet networks.

###  Proposed approach- SWNet model

**Fig. 5:**
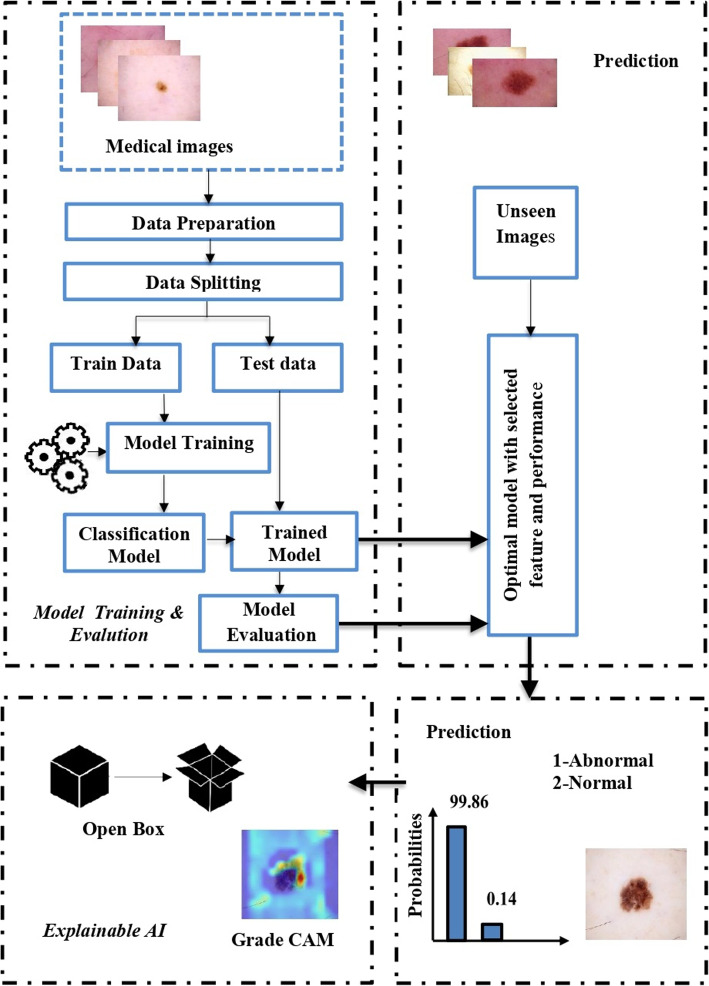
illustrates our classification pipeline

This research paper presents a new design for a deep convolutional neural network called SWNet, which aims to improve the identification of critical elements related to the categorization of melanoma, a type of skin cancer. The system is designed based on a directed acyclic graph (DAG). The classification of melanoma requires a network with a more complex structure to extract more features, which helps to distinguish between normal and abnormal classes. The proposed model has an advantage in expanding its width without increasing computational cost significantly, which increases the amount of information that can be acquired and improves precision. The overall process of the classification methodology is shown in Fig. 5. The SWNet architecture comprises multiple layers, namely:

1. Initially, an Input layer consists of cropping the input image into 224×224 pixels. Cropping the image to a reasonable size helps to retain relevant information while reducing computational overhead and ensuring compatibility with the proposed model, standard, efficient processing, and improved generalizability across different tasks. This task aims to classify these patches into normal and abnormal categories. It makes it possible to analyze and treat the selected regions separately, which can be useful in different computer vision tasks.

2. The convolutional layer is a fundamental component of convolutional neural networks (CNNs). This layer applies convolutions to the output of the preceding layer. The set of filters is learnable because the convolution filter is defined by its weights. The two-dimensional activation maps of the corresponding filters are generated by varying the height and width of the input volume. It is essential to observe that each filter possesses a comparable depth to entries [44]. Furthermore, the output’s dimensions can be controlled by adjusting three hyperparameters: zero padding, stride, and depth. Zero padding involves adding zeroes around the boundaries of the input to maintain its size. Stride pertains to the number of pixels the filter skips across the image. Depth, on the other hand, indicates the quantity of filters applied to the input image. These filters can identify different structures, including blobs, corners, edges, etc. This research utilizes a model of 33 convolutional layers, and increasing the numbe of layers in convolutional neural networks (CNNs) makes it possible to extract hierarchical features, which improves the model’s ability to capture complex patterns. Deeper networks provide better representation and increased ability to learn complex data, each featuring 3 × 3 filters. Following each convolutional layer, subsequent layers of Batch Normalization (BN) and the Rectified Linear Unit (ReLU) are incorporated. 2. The convolutional layer is a fundamental component of convolutional neural networks (CNNs). This layer applies convolutions to the output of the preceding layer. The set of filters is learnable because the convolution filter is defined by its weights. The two-dimensional activation maps of the corresponding filters are generated by varying the height and width of the input volume. It is essential to observe that each filter possesses a comparable depth to entries [44]. Furthermore, the output’s dimensions can be controlled by adjusting three hyperparameters: zero padding, stride, and depth. Zero padding involves adding zeroes around the boundaries of the input to maintain its size. Stride pertains to the number of pixels the filter skips across the image. Depth, on the other hand, indicates the quantity of filters applied to the input image. These filters can identify different structures, including blobs, corners, edges, etc. This research utilizes a model of 33 convolutional layers, and increasing the numbe of layers in convolutional neural networks (CNNs) makes it possible to extract hierarchical features, which improves the model’s ability to capture complex patterns. Deeper networks provide better representation and increased ability to learn complex data, each featuring 3 × 3 filters. Following each convolutional layer, subsequent layers of Batch Normalization (BN) and the Rectified Linear Unit (ReLU) are incorporated. SWNet’s complex architecture achieves high accuracy but comes with significant computational demands, which can be challenging in resource-constrained clinical settings. Trade-offs between model complexity, inference time, and deployment feasibility must be carefully considered. Strategies such as model pruning, quantization, and lightweight versions can optimize SWNet for real-world applications. Balancing performance and computational cost is essential for effective clinical integration.

3. Batch normalization is a method employed in machine learning and deep learning models to normalize the activations of a neural network layer by adjusting and scaling them. This layer is used to normalize every input channel within a mini-batch. This technique accelerates the Convolutional Neural Networks (CNNs) training process and reduces the network initialization’s sensitivity [45]. This study places the batch normalization layer between the convolutional and ReLU layers. This work incorporates 37 batch normalization layers; these layers help stabilize the training process in deeper models. The mechanism of the batch normalization layer involves the normalization of channel activations through the subtraction of the mini-batch average and division by the standard deviation of the mini-batch to enhance stability, elevate learning rates, decrease reliance on explicit regularization, and enhance the management of gradient issues.

4. The Rectified Linear Unit (ReLU) layer filters information by using the max (0, x) function, where x represents the neuron’s input [46]. ReLU benefits neural networks by introducing nonlinearity, addressing the vanishing gradient problem, and providing computational efficiency.

5. The Addition Layer combines the inputs from two or more neural network layers. All inputs must have identical dimensions for this layer to function.

6. The average pooling layer divides its input into rectangular pooling zones of various sizes, such as 2 × 2, 3 x 3, etc. It calculates the average values in each smaller spatial block [47]. Average pooling extracts normalized feature information from significant and insignificant pixel data. Max polling emphasizes edges, corners, and lines to improve an image. In the final stage of the network, we use global average pooling instead of max pooling to avoid losing some attributes to max pooling. All of these qualities, large or small, help differentiate classes.

7. Dropout layers prevent overfitting and improve model performance. This layer activates and deactivates neurons randomly [48]. In our work, two dropout layers are employed between fully connected layers with a dropout probability of *p* = 0.5.

8. Fully Connected (FC) Layer: All of the neurons from the previous layer are connected to all of the neurons in this layer. This layer mixes the attributes that can be used to divide skin patches into two groups: normal and abnormal [47]. Our suggested SWNet comprises three FC layers, which led to performance improvements.

**Fig. 6:**
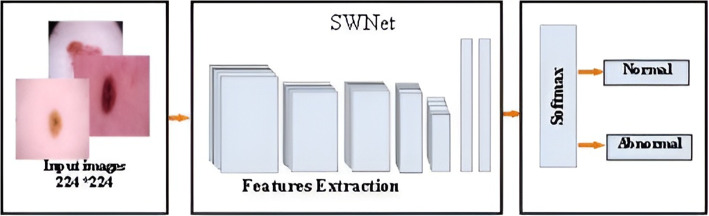
Overview of the network training procedures

**Fig. 7:**
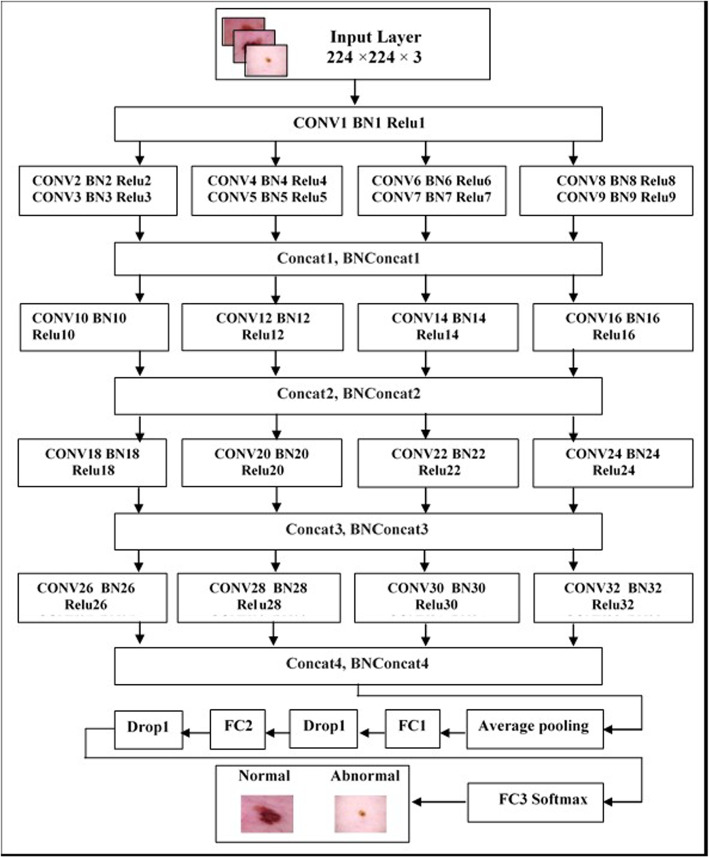
SWNet architecture. Where: CONV = Convolutional layer; BN = Batch Normalization layer; Relu = Rectified Linear Unit layer; Concat = Concatenation Layer (Combines the inputs from two or more neural network layers); BNConcat = Batch Normalization Concatenation layer; Drop = Dropout layer; FC = Fully Connected Layer

Figure 6 demonstrates the utilization of the Softmax function for categorization. The output layer resides at the highest location within the complete and ultimate connected layer, as illustrated in Fig. 7. The total number of SWNet layers is 113. The image classification task requires a deep architecture to better capture complex and essential image patterns through a deeper network. This provided us with better accuracy and performance on other metrics, as shown in Table 1.

SWNet’s architecture, based on CNNs, is highly generalizable and can be adapted to various image classification tasks, including medical and non-medical applications. Its feature extraction techniques, using multiple paths, enhance versatility across different datasets. Robust performance metrics ensure easy adaptation to new tasks, while its compatibility with transfer learning enables leveraging learned features for other domains. These attributes make SWNet a scalable framework beyond skin cancer detection.

**Table 1:** Presents the architectural components of the proposed SWNet model

Name of layer	Kernel size and stride	Activations
Input layer		224×224×3
Conv1, BN1, ReLU1	Conv1: Kernel size=3×3	224×224×32
Conv2, BN2, ReLU2	Conv2: Kernel size=3×3, stride=1	224×224×32
Conv3, BN3, ReLU3	Conv3: Kernel size=3×3, stride=2	112×112×32
Conv4, BN4, ReLU4	Conv4: Kernel size=3×3, stride=1	224×224×32
Conv5, BN5, ReLU5	Conv5: Kernel size=3×3, stride=2	112×112×32
Conv6, BN6, ReLU6	Conv6: Kernel size=3×3, stride=1	224×224×32
Conv7, BN7, ReLU7	Conv7: Kernel size=3×3, stride=2	112×112×32
Conv8, BN8, ReLU8	Conv8: Kernel size=3×3, stride=1	224×224×32
Conv9, BN9, ReLU9	Conv9: Kernel size=3×3, stride=2	112×112×32
Concat1, BNConcat1	Concatenation of four inputs	112×112×128
Conv10,BN10,ReLU10	Conv10: Kernel size=3×3, stride=1	112×112×64
Conv11, BN11, ReLU11	Conv11: Kernel size=3×3, stride=2	56×56×64
Conv12, BN12, ReLU12	Conv12: Kernel size=3×3, stride=1	112×112×64
Conv13, BN13, ReLU13	Conv13: Kernel size=3×3, stride=2	56×56×64
Conv14, BN14, ReLU14	Conv14: Kernel size=3×3, stride=1	112×112×64
Conv15, BN15, ReLU15	Conv15: Kernel size=3×3, stride=2	56×56×64
Conv16, BN16, ReLU16	Conv16: Kernel size=3×3, stride=1	112×112×64
Conv17, BN17, ReLU17	Conv17: Kernel size=3×3, stride=2	56×56×64
Concat2, BNConcat2	Concatenation of four inputs	56×56×64
Conv18, BN18, ReLU18	Conv18: Kernel size=3×3, stride=1	56×56×64
Conv19, BN19, ReLU19	Conv19: Kernel size=3×3, stride=2	28×28×128
Conv20, BN20, ReLU20	Conv20: Kernel size=3×3, stride=1	56×56×128
Conv21, BN21, ReLU21	Conv21: Kernel size=3×3, stride=2	28×28×128
Conv22, BN22, ReLU22	Conv22: Kernel size=3×3, stride=1	56×56×128
Conv23, BN23, ReLU23	Conv23: Kernel size=3×3, stride=2	28×28×128
Conv24, BN24, ReLU24	Conv24: Kernel size=3×3, stride=1	56×56×128
Conv25, BN25, ReLU25	Conv25: Kernel size=3×3, stride=2	28×28×128
Concat3, BNConcat3	Concatenation of four inputs	28×28×512
Conv26, BN26, ReLU26	Conv26: Kernel size=3×3, stride=1	28×28×256
Conv27, BN27, ReLU27	Conv27: Kernel size=3×3, stride=2	14×14×256
Conv28, BN28, ReLU28	Conv28: Kernel size=3×3, stride=1	28×28×256
Conv29, BN29, ReLU29	Conv29: Kernel size=3×3, stride=2	14×14×256
Conv30, BN30, ReLU30	Conv30: Kernel size=3×3, stride=1	28×28×256
Conv31, BN31, ReLU31	Conv31: Kernel size=3×3, stride=2	14×14×256
Conv32, BN32, ReLU32	Conv32: Kernel size=3×3, stride=1	28×28×256
Conv33, BN33, ReLU33	Conv33: Kernel size=3×3, stride=2	14×14×256
Concat4, BNConcat4	Concatenation of four inputs	14×14×1024
Average polling layer		1×1×1024
Fc1	300 fully connected	1×1×300
Drop1	Dropout layer with learning rate:0.5	1×1×300
Fc2	64 fully connected	1×1×64
Drop2	Dropout layer with learning rate:0.5	1×1×300
Fc3 (Softmax layer)	0= Benign, 1= Malignant	1×1×2

**Fig. 8:**
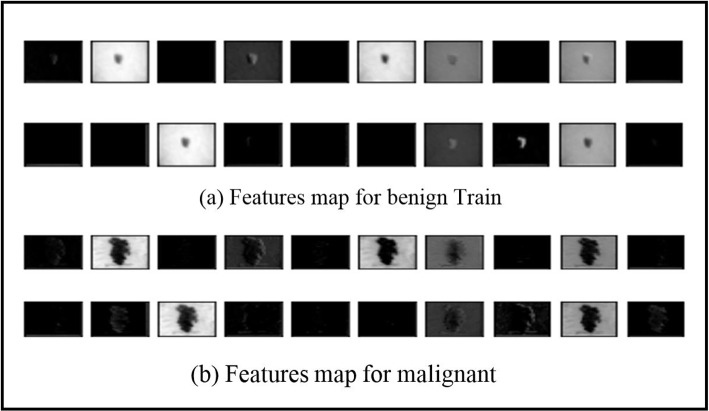
**a**, **b** Features map for benign and malignant

The new SWNet architecture helps to obtain a multi-level advantage at each step. With each wrapping layer, there are more differentiating attributes. Figure 8 shows the activation for both normal and diseased epidermis classes. The model and pre-trained models utilized in this study were trained on a dataset consisting of 10605 images for 100 epochs until the learning process reached a point of convergence. SWNet is a network proven to be the best for classifying skin cancer. SWNet architecture has been built entirely from scratch. For this experiment, we used a RAM of 16 GB and a GPU of 8 GB. The experiments were carried out using Matlab R2023a.

### Feature fusion

Feature fusion is a methodology employed to enhance the effectiveness of machine learning models by combining features sourced from diverse origins. In the specific context of skin cancer detection, this approach proves advantageous as it amalgamates features from disparate datasets of skin cancer images, improving the model’s accuracy. Various methods exist for implementing feature fusion [49–51].

One conventional method entails the simple concatenation of features from different datasets. Although this approach is straightforward, it may need to be more efficient when dealing with datasets that differ in the quantity of features. Alternatively, more advanced techniques like canonical correlation analysis (CCA) or support vector machines (SVMs) can be utilized for feature fusion. These sophisticated methods excel in capturing intricate relationships between features from diverse datasets, ultimately enhancing the model’s overall performance.

**Fig. 9:**
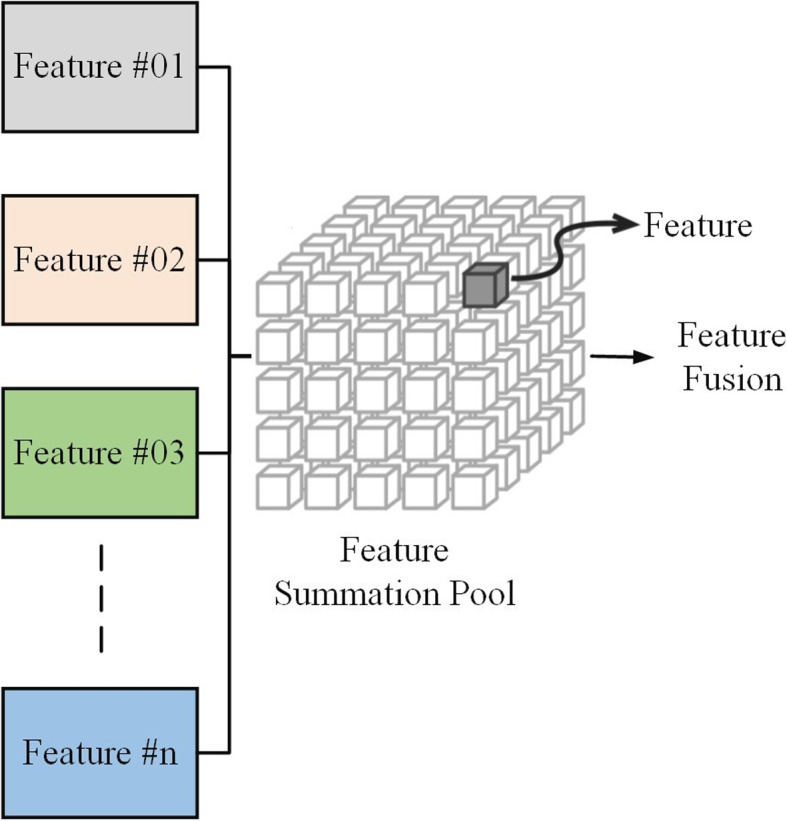
Feature fusion diagram

In skin cancer detection, multi-dataset feature fusion emerges as a promising strategy to address the challenge of data scarcity. The scarcity issue arises due to the limited availability of labeled examples of skin cancer, hindering the training of accurate and adaptable machine learning models for new data. By amalgamating features from multiple datasets, multidataset feature fusion expands the pool of labeled data accessible to the model. This augmentation plays a crucial role in improving both the accuracy of the model and its ability to adapt to new data [52]. Figure 9 shows the fusion structure.

Integrating multi-dataset feature fusion into skin cancer detection brhas several benefits. First, it improves the accuracy of skin cancer detection models. Second, it improves generalizability, allowing these models to perform effectively on new and unseen data. Third, multi-dataset feature fusion addresses overfitting in skin cancer detection models.

However, adopting multi-dataset feature fusion in skin cancer detection is challenging. Ensuring compatibility between datasets in terms of feature representation and labeling poses a significant obstacle. The computational complexity of specific feature fusion methods also presents a potential drawback. Furthermore, interpreting the outcomes of models utilizing multi-dataset feature fusion can be intricate.

Despite these challenges, multi-dataset feature fusion remains a promising avenue for advancing skin cancer detection. With careful design and implementation, it holds the potential to significantly enhance the accuracy and adaptability of skin cancer detection models.

### Explainable artificial intelligence(XAI)

Deep learning networks are commonly characterized as “black boxes” due to their lack of transparency on the underlying rationale behind the network’s decision-making process [53, 54]. The utilization of deep learning networks is increasingly prevalent across numerous disciplines, including medical care, so understanding why the network makes a particular decision is critical.

A subset of interpretability techniques called visualization methods uses visual illustrations to describe what the network is looking at and its predictions. This topic focuses on post-training techniques that annotate predictions made by a network trained on image data using test images [55, 56].

**Fig. 10:**
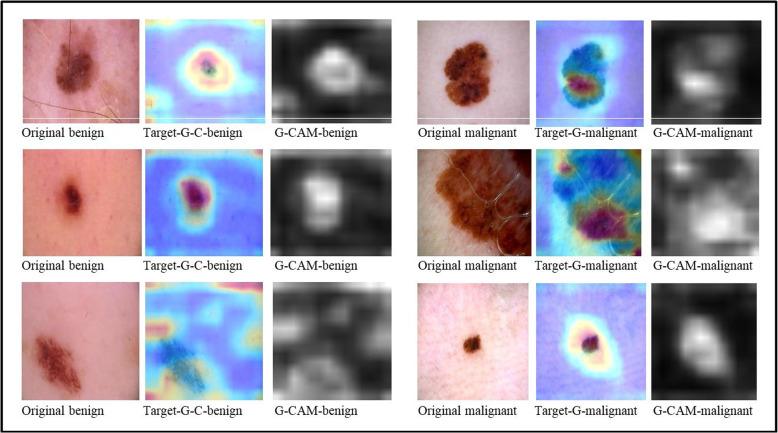
Images taken using Grad-CAM

We incorporated interpretable artificial intelligence (XAI) techniques into our proposed model to ensure transparency and interpretability. Specifically, we used Grad-CAM to identify features of interest, generate interpretations of the trained model’s predictions, and evaluate model reliability. The Grad-CAM heatmap is a visual representation that identifies the specific regions within an image that have the most significant influence on the prediction of a target. By superimposing a Grad-CAM heat map onto the original image, it becomes possible to observe how parts of the image or input data that influence the model’s decision are highlighted and to assign relevance scores to different features by analyzing the gradients or heat maps produced by Grad-CAM, thus giving us a clear understanding of the model’s predictions. The classification score hierarchy concerning convolutional features generated by the network is used to ascertain which regions of the image hold the highest importance for classification purposes. The following Fig. 10 illustrations are part of our target applications for test images by using Grad-CAM for 9 layers.

## Experimental result

The dataset was partitioned into two phases: a training phase and a testing phase. We conducted a series of experiments on the dataset to evaluate the classification performance of our network, as well as the fine-tuned networks that were employed. Applying interpretable artificial intelligence (XAI) techniques significantly improved the interpretability of our proposed model. The generated interpretations allowed us to identify the main factors that influence the model predictions and understand their reasons. By analyzing the explanations provided, we could confirm the model’s consistency with domain knowledge and identify potential areas for improvement.

An F1 score is used to evaluate the performance of the proposed and finetuned models. F1_score represents the balance between recall (R) and precision (P), two crucial characteristics for assessing the proposed technique. Precision, recall, and F1_score are computed using Eqs. (1), (2), and (3).

1 $$\begin{aligned} \text {Precision} = \frac{\text {TP}}{\text {TP} + \text {FP}} \end{aligned}$$

2 $$\begin{aligned} \text {Recall} = \frac{\text {TP}}{\text {TP} + \text {FN}} \end{aligned}$$

3 $$\begin{aligned} \text {F1\_Score} = \frac{\text {Precision} \times \text {Recall}}{\text {Precision} + \text {Recall}} \end{aligned}$$

**Fig. 11:**
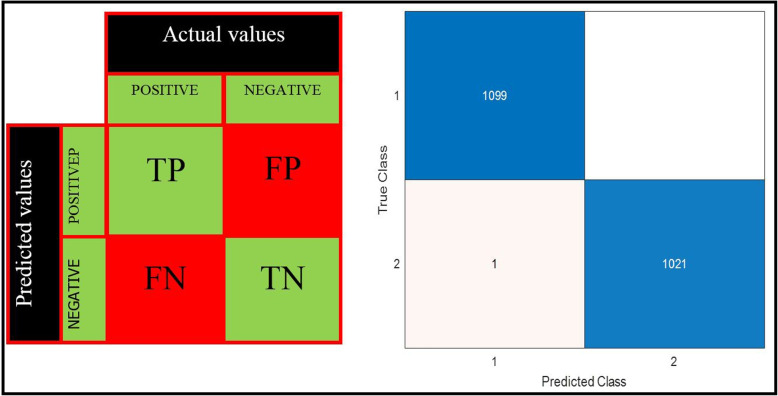
The proposed confusion matrix of SWNet

The number of images that the network correctly classifies as relevant is TP (true positive). The number of images that the network correctly classifies as irrelevant is TN (True Negative). The number of images that the network mistakenly classifies as relevant is FP (False Positive). The number of relevant images that the network cannot recognize is FN (False Negative), as seen in the general confusion matrix Fig. 11a, and b represents our data confusion matrix.

**Fig. 12:**
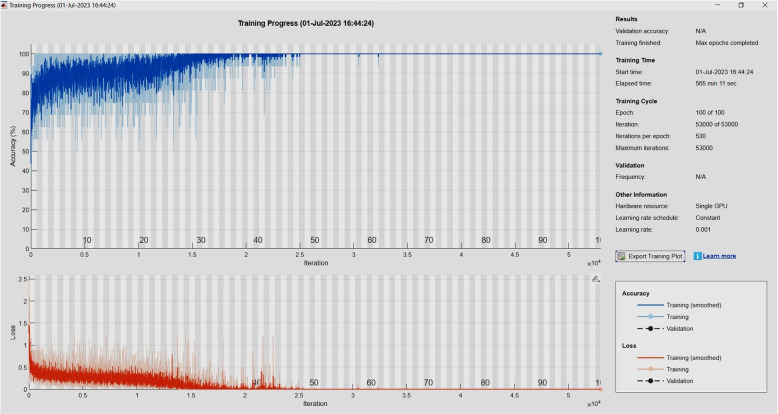
Illustration of training process

The model was trained using the SGD optimizer with a learning rate 0.001 and other specified hyperparameters such as batch size. The optimal outcome was reached by executing 100 epochs, resulting in an accuracy metric of 99.86%. Figure 12 illustrates the process of our training.

In Table 2, a comparison is presented between the current study and previous research. The metrics of the proposed SWNet were reported using the Softmax classifier, demonstrating its superior performance. SWNet achieved the highest standards of precision, recall, and F1-score at 100%, 99.90%, and 99.95%, respectively. These results conclude that SWNet outperforms existing models as a robust tool for skin cancer detection.

**Table 2:** Comparison of SWNet with similar studies

Net work	Year	Precision	Recall	F1-Score	Accuracy
S. Waheed et at. [30]	2023	92.46%	92.46%	90.87%	91%
Y. Dahdouh, et al. [31]	2023	-	-	-	80%
M. Tahir, et al. [28]	2023	94.17	93.76	93.93	94.28
Proposed SWNet	2024	100%	99.90	99.95	99.86

**Fig. 13:**
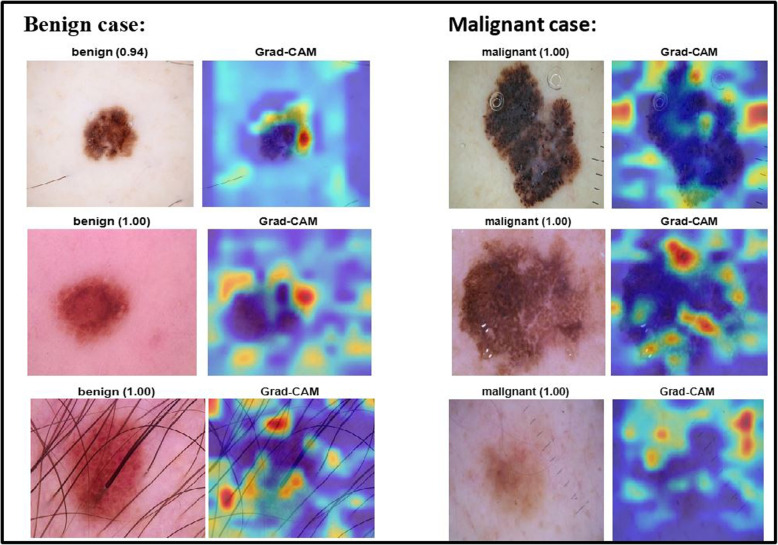
Predictions of test samples by SWNet enhanced with explainable artificial intelligence (XAI)

Figure 13 displays the prediction of some test images using SWNet with Explainable AI (XAI) technique. Table 3 compares SWNet with contemporary networks for classification, assessing their performance across various metrics. The evaluated networks include EfficientNet, MobileNet, Darknet, and the proposed SWNet. The metrics considered are Precision, Recall, Specificity, F1-Score, and Accuracy, providing a holistic view of the classification capabilities of each network.

EfficientNet achieves a Precision of 89.05%, recall of 89.77%, Specificity of 88.03%, F1-Score of 86.39%, and Accuracy of 91.88%. MobilNet follows closely with a Precision of 89.64%, recall of 90.22%, specificity of 87.60%, F1-Score of 92.23%, and an Accuracy of 93.2%. Darknet outperforms both with a Precision of 91.66%, Recall of 93.10%, specificity of 90.18%, F1-Score of 92.17%, and an Accuracy of 94.44%.

Notably, the proposed SWNet surpasses all, achieving exceptional results across all metrics. SWNet attains a perfect precision of 100%, an impressive recall of 99.90%, a perfect specificity of 100%, an outstanding F1-Score of 99.95%, and an accuracy of 99.86%. These results highlight the superior classification performance of SWNet compared to the other modern networks, making it a compelling choice for the task.

SWNet’s perfect precision and specificity indicate its ability to minimize false positives, which is essential in applications where misclassifying a positive instance is critical. The high recall underscores its effectiveness in capturing true positive instances, while the remarkable F1-Score reflects a balance between Precision and Recall. The overall high accuracy further solidifies SWNet’s position as a robust and reliable choice for classification tasks, outperforming well-established networks in the field. In summary, SWNet sets a new benchmark for classification tasks, making it a compelling choice for applications requiring high precision, reliability, and interpretability.

**Table 3:** Comparison of SWNet with modern networks for classification

Model	Precision	Recall	Specificity	F1-Score	Accuracy
EfficientNet [42]	89.05	89.77	88.03	86.39	91.88
Mobilnet [37]	89.5	90.22	87.60	92.23	93.2
Darknet [43]	91.66	93.10	90.18	92.17	94.44
Proposed SWNet	100	99.90	100	99.95	99.86

The provided Table 4 presents a comparative analysis of several neural network models based on various parameters. Each model is assessed according to its number of convolution layers, the quantity of convolution kernels employed, the presence of pooling layers, the utilization of batch normalization, the count of fully connected layers, and the recognition time. EfficientNet showcases a comprehensive architecture with its 65 convolution layers and a substantial number of convolution kernels (3,328,992). It incorporates 17 pooling layers, utilizes batch normalization, and boasts 49 fully connected layers, achieving a recognition time of 15 milliseconds. In contrast, MobileNet features fewer convolution layers (15) and a moderate number of convolution kernels (4,253,864), with only one pooling layer and no batch normalization. Despite its simpler architecture, MobileNet demonstrates a shorter recognition time of 10 milliseconds. Darknet, with 18 convolution layers and a significant number of convolution kernels (19,810,176), emphasizes batch normalization across its architecture. However, it exhibits a longer recognition time of 25 milliseconds. Finally, the proposed SWNET model introduces 33 convolution layers and a notable number of convolution kernels (9,368,192), along with batch normalization and 37 fully connected layers, resulting in a recognition time of 20 milliseconds. These specifications offer insights into theach model’s computational complexities and inference speeds enabling informed decisions regarding their suitability for specific applications.

**Table 4:** Models parameters where N1: No. convolution layers; N2: No. convolution kernels; N3: No. pooling layers; N4: No. Batch normalization; N5: No.fully connected layer: N6: Recognition Time; GAP: Global Average Pooling

Network	N1	N2	N3	N4	N5	N6
EfficientNet [42]	65	3328992	17 (GAP)	49	1	15 ms
MobilNet [37]	15	4253864	1 (GAP)	27	0	10 ms
Darknet [43]	18	19810176	6 (Max pooling)	18	1	25 ms
Proposed SWNet	33	9368192	1 (GAP)	37	3	20 ms

On the other hand, we conducted several experiments to test the proposed model on different sets of databases to compare performance. The results are shown in Table 5.

**Table 5:** Perforance comparsion of the used dataset in our experiment

Dataset	Acc.	Sen.	Spe.	Pre.	F1-Score	# Images
Mnist-ham10000 [57]	81.93	55.73	85.71	36.06	43.79	10015
ISIC2019 [58]	84.76	84.08	85.39	84.08	84.08	2099
ISIC2019_2020 [59]	91.09	92.60	90.01	86.97	89.70	11449
Melanoma Skin [60]	99.86	99.90	100	100	99.95	10605

Table 5 presents the outcomes of our proposed method using the mnist-HAM10000 dataset, revealing an accuracy of 81.93%. Despite having the most significant number of training images among the datasets, this accuracy may not suffice for specialists to rely on, as indicated by the low F-score of 43.79%, signifying an imbalance in the data. The model exhibits bias towards the class with a higher volume of data. Conversely, with the ISIC2019 dataset containing a smaller training set of 2357 images, the accuracy improves to 84.76%. However, in experiments with the ISIC-2019–2020 melanoma dataset, the model achieves an accuracy of 91.09% and an F-score of 89.70%, surpassing previous results. Despite this improvement, the model needs to meet the required accuracy for diagnosing diseases due to the prevalence of darkness in the background of trained photomicrographs. Nevertheless, the proposed SWNet model demonstrates superior performance in early skin cancer detection, successfully discerning complex differences between benign and malignant behaviors, and showcasing its effectiveness across multiple databases.

### Skin cancer bias mitigation in dataset

**Fig. 14:**
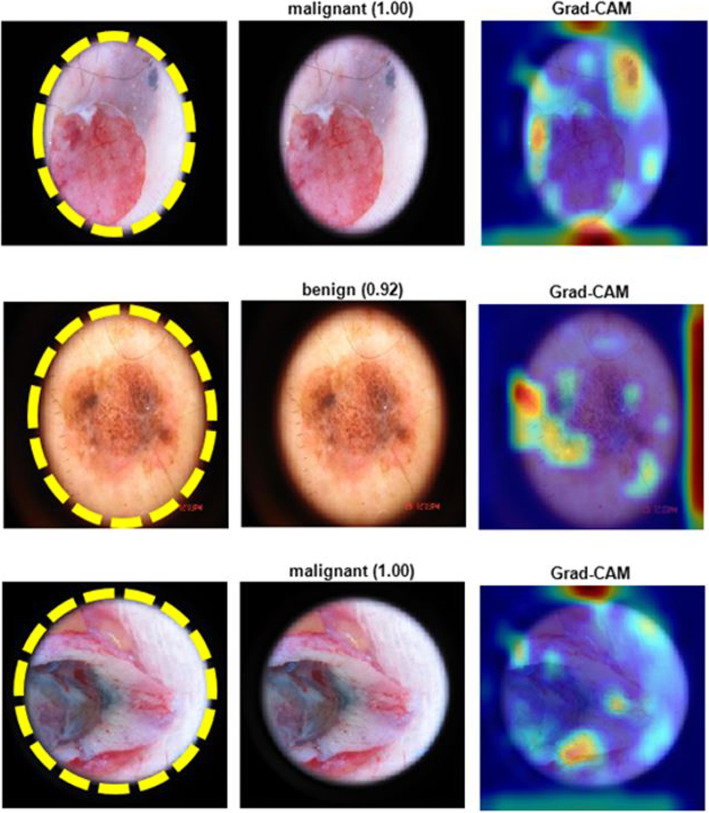
The initial column displays the ground truth images, the second column showcases the outcomes post-classification, and the third column exhibits the Grad-CAM visualizations

The Skin Cancer dataset poses a potential risk of bias in AI models, as seen in Fig. 14, explicitly affecting the SWNet model’s interpretation of darkness as a circular image, a consequence of microscope artefacts. This bias has the potential to impede the model’s ability to generalize effectively, creating challenges in adapting to the diverse and non-circular patterns characteristic of real-world skin abnormalities. Additionally, the dataset’s limitations in adequately representing various skin types, ethnicities, and conditions further undermine the model’s robustness across diverse populations, giving rise to concerns regarding potential misdiagnoses and oversimplification of the nuanced manifestations of skin cancer. To address these challenges, a diverse dataset is necessary to represent different skin types, ethnicities, and conditions. This inclusivity is essential for fostering a more comprehensive learning process. Augmenting the dataset through rotation and scaling becomes imperative to artificially introduce diversity, thereby reducing the model’s sensitivity to specific features like circular patterns resulting from microscope artefacts. Another critical strategy involves incorporating adversarial training during the model development phase. This proactive approach exposes the model to potential biases, promoting adaptability and reducing susceptibility to artefacts. Regular model audits and continuous monitoring post-deployment are essential to promptly identify and rectify emerging biases, guaranteeing the model’s reliability in real-world scenarios. Furthermore, integrating Explainable AI (XAI) techniques contributes to transparency, enabling healthcare professionals to understand the model’s decision-making process. Collaboration with domain experts, particularly dermatologists and healthcare professionals, proves invaluable. Their expertise validates the model’s outputs and enhances its clinical relevance and accuracy. Establishing feedback loops for real-world performance data and implementing iterative model improvement processes become crucial. This dynamic approach empowers developers to address evolving challenges and maintain the model’s effectiveness. Lastly, a steadfast commitment to ethical considerations, focusing on fairness, equity, and patient well-being, guides AI’s responsible development and deployment in healthcare. This commitment fosters trust among users and ensures positive outcomes in medical applications.

Integrating feature fusion from diverse datasets, including Mnist-ham10000, ISIC2019, ISIC2019_2020, and Melanoma Skin Cancer, presents a comprehensive approach to enhancing the performance of risk-biased skin cancer classification. Combining unique features extracted from these varied sources gives the model a more nuanced understanding of skin abnormalities, thereby improving its ability to discern risks associated with different skin conditions. The Mnist-ham10000 dataset contributes information about diverse skin types and conditions, while the ISIC2019 and ISIC2019_2020 datasets offer insights into a broad spectrum of skin lesions. Including the Melanoma Skin Cancer dataset further enriches the feature set with specific details pertaining to melanoma, a critical aspect in risk assessment. Through feature fusion, this amalgamation of datasets not only broadens the model’s knowledge base but allows it to adapt to the intricacies of diverse skin characteristics. The summation of features from these datasets collectively fortifies the risk-biased skin cancer classification model, fostering improved accuracy and reliability in identifying potential health risks across a wide range of skin conditions and patient demographics.

**Fig. 15:**
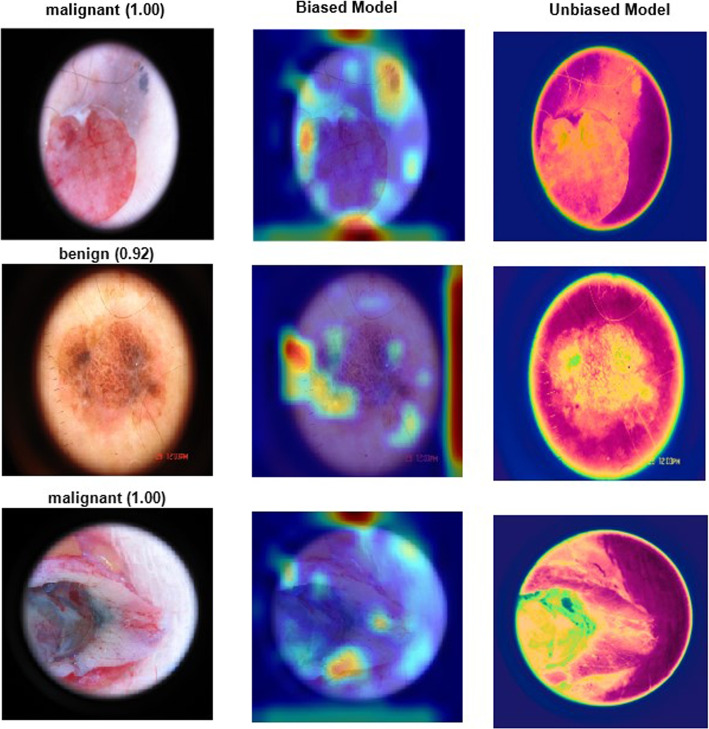
Feature fusion: The first column presents the outcomes after classification, the second column highlights a biased model, and the third column demonstrates the results of the unbiased model

Feature fusion emerges as a potent strategy for addressing bias and promoting fairness within skin cancer datasets by incorporating diverse features, as seen in Fig. 15. Integrating features from various datasets, including Mnist-ham10000, ISIC2019, ISIC2019_2020, and Melanoma Skin Cancer, contributes to a more extensive and inclusive understanding of skin conditions. This method alleviates bias by exposing the model to a broader spectrum of skin types, lesions, and conditions, reducing the likelihood of favouring specific demographics. The potential bias from a limited dataset can be countered by incorporating features related to diverse skin characteristics, demographic information, and lesion types through feature fusion. This enables the model to identify patterns across various skin types, ethnicities, and conditions, ensuring a more comprehensive and equitable performance. As a result, the precision of skin cancer classification is improved, and the model’s predictions remain impartial and unbiased across diverse patient populations.

Furthermore, incorporating features from multiple datasets enables the model to adapt to the intricacies of real-world scenarios, effectively addressing concerns about fairness. For example, features from the Melanoma Skin Cancer dataset specifically focus on melanoma, providing vital information for accurate risk assessment in cases of this particular skin condition. The holistic perspective facilitated by feature fusion contributes to a more nuanced understanding of skin abnormalities, promoting fairness in the model’s predictions. Therefore, feature fusion serves as a mechanism to reduce bias in skin cancer datasets by introducing a diverse array of features, fostering a more balanced and representative learning experience for the model. This enhances the model’s overall performance and ensures its predictions are fair, unbiased, and applicable across a broad spectrum of skin conditions and patient demographics.

**Fig. 16:**
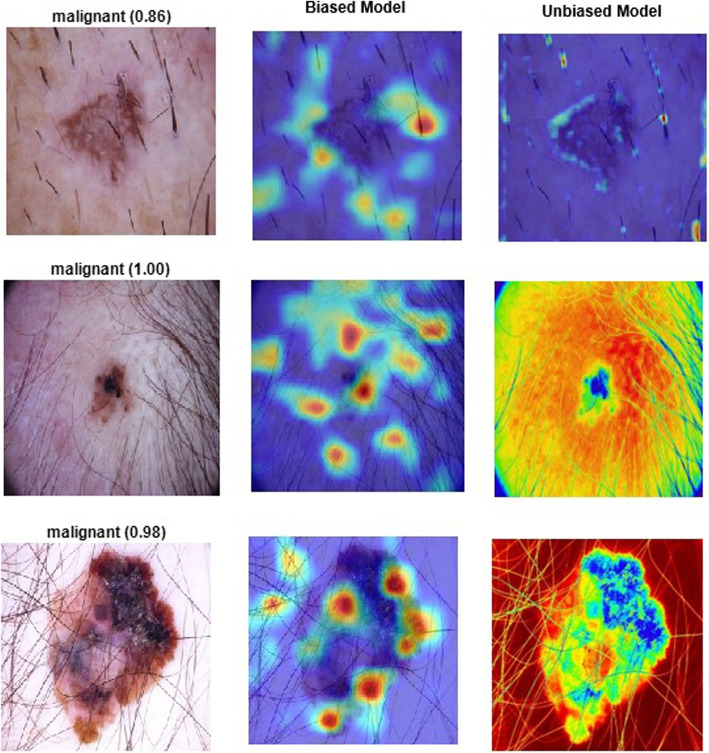
Challenges in hair-related bias

Figure 16 shows that biases can arise in hair skin cancer datasets due to the challenges posed by hair. These biases are especially prevalent in individuals with darker skin tones or excessive hair. It is crucial to incorporate feature fusion and assimilate insights from various datasets to gain a more comprehensive understanding of the factors that influence skin health, including hair coverage.

Incorporating features linked to hair coverage improves the model’s ability to distinguish between skin abnormalities and hair patterns. This targeted inclusion addresses the unique challenges of hair skin cancer datasets, ensuring the model is better equipped to navigate the complexities introduced by the presence of hair. This enhances the accuracy of skin cancer detection and fosters fairness by minimizing biases associated with hair coverage.

Furthermore, the diverse features introduced through feature fusion help alleviate biases tied to demographic factors and environmental conditions, fostering a more inclusive and equitable model. The model’s ability to adapt to a broad range of features, including those associated with hair, contributes to a fair and reliable skin cancer detection framework that accommodates the diverse characteristics of individuals, regardless of hair coverage or skin type. Feature fusion is a powerful tool to counter biases and enhance fairness in hair and skin cancer datasets by enriching the model’s knowledge with a comprehensive set of pertinent features.

## Limitations and future directions

The implementation of the SWNet model for skin cancer detection faces several limitations that need to be addressed:

1. Data Storage and Management: Handling a large set of high-quality or large-sized images poses significant challenges in terms of loading, storing, and processing the data efficiently.
2. Optimization Complexity: As data size increases, optimizing model parameters, such as the learning rate and batch size, becomes increasingly complex, requiring meticulous fine-tuning to achieve optimal performance.
3. Dependency on Preprocessing Techniques: The performance of the SWNet model is highly dependent on the effectiveness of the preprocessing techniques used to extract features. Limitations in these techniques can adversely affect the model’s overall performance.

To overcome these challenges and further advance the capabilities of the SWNet model, several future research directions can be pursued:

1. Utilization of Real-World Clinical Datasets: Future research should prioritize leveraging datasets from real-world clinical settings to better evaluate the model’s performance and robustness. This would provide valuable insights into the model’s applicability in practical scenarios.
2. Exploration of Advanced Features and Techniques: Investigating additional handcrafted features or integrating other deep learning techniques can further enhance the model’s performance. This includes experimenting with alternative architectures and feature extraction methods.
3. Addressing Underrepresented Classes: Developing more advanced techniques to improve the model’s performance on underrepresented classes is crucial for achieving balanced and accurate detection.
4. Real-Time Clinical Deployment: Developing real-time skin cancer detection systems optimized for speed and efficiency, without compromising accuracy, would enable their deployment in clinical settings, offering practical benefits to healthcare providers and patients.
5. Longitudinal Performance Evaluation: Conducting longitudinal studies to assess the model’s performance over time and its ability to adapt to new data can provide insights into its long-term reliability and effectiveness in clinical practice.

## Conclusions

We introduced SWNet, a novel convolutional neural network designed for the automated classification of skin cancer into benign and malignant categories. SWNet’s architecture strategically enhances network width, offering significant advantages without escalating computational costs. Feature fusion was incorporated during model training on a public dataset, effectively addressing potential biases associated with skin conditions, particularly in individuals with darker skin tones or excessive hair.

The extracted features were used to train the SoftMax classifier layer. To improve interpretability, we integrated explainable artificial intelligence (XAI) methodologies, specifically Grad-CAM, to identify salient features, generate interpretations of predictions, and assess the model’s reliability. This approach provided valuable insights into the decision-making process, fostered a deeper understanding, and enhanced confidence in the model’s outputs.

Comparative analysis with state-of-the-art networks, including EfficientNet, MobileNet, and Darknet, pre-trained on the ImageNet dataset, demonstrated SWNet’s superiority. The model achieved an accuracy of 99.86% and an F1 score of 99.95%, surpassing these benchmarks. Furthermore, SWNet’s ability to classify normal and abnormal classes and its integration of feature fusion to mitigate biases reinforce its robustness and reliability in addressing diverse skin conditions.

## Data Availability

We have used public datasets as follows: 1-Mnist-ham10000 Dataset: https://kaggle.com/datasets/kmader/skin-cancer-mnist-ham10000. 2- ISIC2019 Dataset: https://kaggle.com/datasets/nodoubttome/skin-cancer9-classesisic. 3- ISIC 2020 Dataset: https://kaggle.com/datasets/qikangdeng/isic-2019-and-2020-melanoma-dataset. 4- Melanoma Skin Cancer Dataset: https://kaggle.com/datasets/hasnainjaved/melanoma-skin-cancer-dataset-of-10000-images.
